# The Microbiome Composition of a Man's Penis Predicts Incident Bacterial Vaginosis in His Female Sex Partner With High Accuracy

**DOI:** 10.3389/fcimb.2020.00433

**Published:** 2020-08-04

**Authors:** Supriya D. Mehta, Dan Zhao, Stefan J. Green, Walter Agingu, Fredrick Otieno, Runa Bhaumik, Dulal Bhaumik, Robert C. Bailey

**Affiliations:** ^1^Division of Epidemiology and Biostatistics, School of Public Health, University of Illinois at Chicago, Chicago, IL, United States; ^2^Sequencing Core, College of Medicine, University of Illinois at Chicago, Chicago, IL, United States; ^3^Nyanza Reproductive Health Society, Kisumu, Kenya

**Keywords:** penile microbiome, penile microbiota, circumcision, bacterial vaginosis, machine learning, ensemble voting, synthetic minority oversampling technique, Kenya

## Abstract

**Background:** We determined the predictive accuracy of penile bacteria for incident BV in female sex partners. In this prospective cohort, we enrolled Kenyan men aged 18–35 and their female sex partners aged 16 and older. We assessed BV at baseline, 1, 6, and 12 months. Incident BV was defined as a Nugent score of 7–10 at a follow-up visit, following a Nugent score of 0–6 at baseline. Amplification of the V3–V4 region of the bacterial 16S rRNA gene was performed on meatal and glans/coronal sulcus swab samples. Majority vote classifier combined the decisions of three machine learning classification algorithms (Random Forest, Support Vector Machine, K Nearest Neighbor). We report the estimate cross-validation predictive accuracy for incident BV based on baseline penile taxa.

**Results:** The incidence of BV was 31% among 168 couples in which the woman did not have BV at baseline: 37.3% if the man was uncircumcised vs. 26.3% if the man was circumcised. Incident BV occurred at 1 month (*n* = 23), 6 months (*n* = 20), 12 months (*n* = 9). The predictive capacity of meatal taxa was high: sensitivity (80.7%), specificity (74.6%), accuracy (77.5%), area under the curve (88.8%). Variable importance ranking identified meatal taxa that in the vagina are associated with BV: *Parvimonas, Lactobacillus iners, L. crispatus, Dialister, Sneathia sanguinegens*, and *Gardnerella vaginalis* were among the top 10 most predictive taxa. The accuracy of glans/coronal sulcus taxa to predict incident BV was comparable to meatal taxa accuracy, but with greater variability.

**Conclusions:** Baseline penile microbiota accurately predicted BV incidence in women who did not have BV at baseline, with more than half of incident infections observed at 6- to 12- months after penile microbiome assessment. These results suggest interventions to manipulate the penile microbiome may reduce BV incidence in sex partners, and that potential treatment (antibiotic or live biotherapeutic) will need to be effective in reducing or altering bacteria at both the glans/coronal sulcus and urethral sites (as represented by the meatus). The temporal association clarifies that concordance of penile microbiome with the vaginal microbiome of sex partners is not merely reflecting the vaginal microbiome, but can contribute to it.

## Introduction

Bacterial vaginosis (BV) is a condition of public health concern, affecting 20–50% of the general population of women in sub-Saharan Africa (Torrone et al., 2018), and associated with increased risk of HIV acquisition and other sexually transmitted infections (STI) (Atashili et al., 2008; Lewis et al., 2017). In pregnant women, BV increases risk of preterm delivery, preterm labor, and late miscarriages (Leitich and Kiss, 2007). Treatment with long-term efficacy is lacking and BV recurs in up to 50% of women at 6–12 months following treatment (Bradshaw and Sobel, 2016). Epidemiologic and microbiologic evidence suggests a role of male sex partners in women's risk of BV and recurrence (Mehta, 2012). For example, female sex partners of Ugandan men undergoing voluntary medical male circumcision (VMMC) had 40% lower prevalence of BV at 1 year (Gray et al., 2009).

Circumcised men have lower presence and abundance of penile anaerobic bacteria (Price et al., 2010; Mehta et al., 2012), several of which are associated with BV. Characterization of the coronal sulcus microbiome among Ugandan men undergoing VMMC identified specific community state types (CSTs) associated with BV in female sex partners (Liu et al., 2015). The reduction in BV associated with male circumcision implies that these bacteria are harbored in the inner foreskin, or that the sub-preputial space makes the glans and/or coronal sulcus a hospitable environment for anaerobic bacteria associated with BV. However, despite reduced BV following male partner circumcision, women with a circumcised male partner still have BV: in the Ugandan study, the prevalence of BV at 1 year among women with circumcised partners was 40.3% (Gray et al., 2009). The urethra or semen may be other reservoirs of bacteria that may affect female partner risk of BV. Nelson et al. characterized the microbiota of urine to approximate the potential microbiota of the urethra, and found that while it was distinct from coronal sulcus composition, it contained high abundances of bacteria that are also found in the vagina (Nelson et al., 2012). Among monogamous heterosexual couples, Zozaya et al. showed that the vaginal taxa were strongly correlated with male partner's penile taxa among couples where the woman had BV, but the correlations were substantially weaker in couples where the women did not have BV (Zozaya et al., 2016). Mändar et al. found a high concordance of microbiota between semen and vaginal samples, supporting their hypothesis that “semen serves as a medium for the transmission of microorganisms between men and women” (Mandar et al., 2015). These studies provide strong evidence for the sexual exchangeability of the vaginal and penile microbiota in relation to BV, and demonstrate that there are multiple male genitourinary reservoirs for these taxa. However, they do not demonstrate temporality of the association between penile microbiome composition and *incident* BV or whether one penile reservoir may be more relevant than others.

The goal of our study was to determine the capacity of bacteria recovered from the meatus of circumcised and uncircumcised men to predict incident BV in female sex partners. Establishing the temporal association is necessary to better quantify the potential contribution of the penile microbiome to *development* of BV. Additionally we sought to assess whether the predictive capacity or specific taxa were similar or different in a subset of men with measurement from the glans/coronal sulcus. Information from both penile sites is useful to understand whether different sites of penile microbiota carry different predictive capacity for BV and whether taxa associated with BV vary by penile location, which may have implications for potential therapeutic choices.

## Materials and Methods

This study was approved by the Ethical Review Committee of Maseno University (Kisumu, Kenya; MSU/DRPC/MUERC/00054/13; January 13, 2014), and the Institutional Review Board of the University of Illinois at Chicago (USA; 2013-0511; February 12, 2014).

### Study Design and Participants

This study of the penile microbiota used data and biological specimens from *Afya Jozi, Afya Jamii* (Kiswahili for “Healthy Pair, Healthy Community”), a prospective cohort study of heterosexual couples in Kisumu, Kenya. Recruitment and eligibility criteria have been published (Mehta et al., 2018). Briefly, to be eligible, members of couples independently confirmed they had been in a sexual relationship for at least 6 months, and agreed to attend all study visits together. We included men ages 18–35 years and their female partners ages 16 years and older. We excluded couples if a member was participating in another research study. Men or women reporting antibiotic use within the past 60 days, men who were circumcised within the past 6 months, and women who reported vaginal douching within the past 7 days were temporarily excluded until sufficient time had passed. Those with multiple sex partners were asked to bring their “main” sex partner. After the baseline visit, couples were scheduled for follow-up at 1, 6, and 12 months. Couples included in this analysis were enrolled between April 1, 2014, and June 22, 2016; all 12-month follow-up visits had been completed by June 21, 2017.

### Clinical Procedures and Follow-Up

Following written informed consent, at baseline and each scheduled follow-up visit, men and women underwent study procedures separately in private examination rooms. At each visit, participants underwent a standardized medical history and physical examination and personal interview to obtain socio-demographic information and information on sexual behavior. Trained clinicians and counselors who were the same sex as the participant interviewed participants in their language of choice (English, Dholuo, or Kiswahili).

### Detection of Bacterial Vaginosis

At baseline and each follow-up visit, a vaginal swab was collected by the female clinician for assessment of BV. Specimens were taken to the on-site lab, and immediately heat-fixed for subsequent Gram staining. Each Gram stained slide was evaluated for morphotypes according to Nugent's criteria (Nugent et al., 1991), and summed to yield a score from 0 to 10. A score of 7–10 was defined as BV. Clinical treatment was provided at point of care and based on detection of three or more of Amsel's criteria in a second vaginal swab (Amsel et al., 1983).

### Penile Specimen Collection

At baseline and each follow-up visit, two penile swabs were obtained by a male clinician from each male participant: (1) meatal swab; (2) glans/coronal sulcus swab. Clinicians used pre-moistened mini-tip flocked swabs (Copan Diagnostics, Inc., Corona, California, USA). To obtain the meatal swab, the clinician twirled the swab around the meatal opening for 3–5 times. To obtain the glans/coronal sulcus swab, clinicians rolled the swab completely around the glans 3 times while continuously twirling the swab, attempting to cover the height of it; the same swab was twirled around the coronal sulcus twice, and then twice around the distal shaft just under the coronal sulcus. In uncircumcised men, the foreskin was retracted prior to sampling. Swabs were immediately placed in the collection kit tube and stored at −80°C until shipment to Chicago for processing. Meatal swabs were collected from all men at all study visits. Glans/coronal sulcus swabs were collected from men for the first 7 months of the study (April 2, 2014 through October 30, 2014).

### Sample Data Used in This Analysis

Of 211 couples with paired longitudinal measures, 43 (20%) had BV (Nugent score 7–10) at baseline and data from these couples are excluded from this analysis of incidence. Therefore, this analysis represents 168 couples in which the woman did not have BV at baseline and in whom at least one follow-up visit was available to determine incidence, and in which the man's baseline penile microbiome measure was complete. In these 168 couples, the median number of follow-up visits was 3, and 78 couples (46%) completed all 4 visits: 1-month follow-up (89%), 6-month follow-up (73%), 12-month follow-up (55%).

### Characterization of Microbial Community Structure

DNA extraction from swabs was performed using EZ1 instrument, implementing the EZ1 DNA tissue protocol (Qiagen, Hilden, Germany). Genomic DNA (gDNA) recovered from penile swabs was used as template for PCR amplification of the V3–V4 variable region of bacterial 16S rRNA gene. A two-step PCR protocol was performed employing the primers 341F and 806R containing Fluidigm AccessArray for Illumina CS1 and CS2 linkers, as described previously (Naqib et al., 2018) with the exception that 2X AccuPrime SuperMix II (Life Technologies, Gaithersburg, MD) was used for PCR amplification. Amplicons were sequenced on an Illumina MiSeq instrument, implementing V3 chemistry (600 cycles) and custom Fluidigm sequencing primers.

Sequence processing and annotation was conducted by University of Maryland Institute for Genomic Science (UMD IGS). Briefly, reads were assembled as described previously (Holm et al., 2019), and amplicon sequence variants (ASVs) generated by DADA2 were individually taxonomically classified using the RDP naïve Bayesian classifier (19) trained with the SILVA v128 16S rRNA gene sequence database (20). ASVs of major vaginal taxa were assigned species-level annotations using speciateIT (version 2.0), a rapid per-sequence classifier (http://ravel-lab.org/speciateit/), and verified via BLASTn against the NCBI 16S rRNA gene sequence reference database. Read counts for ASVs assigned to the same taxonomy were summed, and a biological observation matrix was generated at the lowest taxonomic level identifiable. We included seven DNA extraction controls and seven blank PCR controls. These samples generated very few reads (range of 0–75 clusters per blank across six blanks), while those of the samples were median 25,997 sequences/sample (interquartile range: 19,524–33,779). Due to the low level of detected contamination, the negative control samples were not included in further analysis of the project, and we did not take steps to remove contaminant reads from our analyses. Data were filtered separately by anatomic site to retain taxa that contributed at least 0.01% of the total sequence reads. This resulted in selection of 54 taxa for the meatal samples and 49 taxa for glans/coronal sulcus samples. Raw sequence data files are available in the Sequence Read Archive (National Center for Biotechnology Information; BioProject identifier PRJNA 516684).

### Data Preparation

Due to the sparsity and compositional nature of high-throughput sequencing datasets, sequence count data were transformed using centered log-ratio (CLR) method prior to statistical analysis (Gloor et al., 2017). Prior to CLR transformation, observations with 0 sequence counts in a particular taxon were imputed by generating a uniform distribution (0.5, 0.95). In general, machine learning algorithms objective functions will not work efficiently without normalization. Since the range of values of CLR transformed data varies widely, normalizing the range of features ensures that each feature contributes approximately proportionately to the final distance. Therefore, subsequent to CLR transformation, data were then normalized for each taxon across all subjects:

$$\begin{array}{l} x_{new}=\frac{x-x_{min}}{x_{max}-x_{min}}. \end{array}$$

After normalization, all the data were scaled from 0 to 1, and thus less sensitive to parameter tuning.

We examined the capacity of the penile microbiome at baseline to predict incident BV among women who did not have BV at baseline. The outcome for analysis was first incident BV, occurring in 31% of the 168 couples for which baseline meatal samples were used. This class imbalance in the rate of outcome results in classification that is biased toward classifying the majority class (He and Garcia, 2009) (i.e., observations which remain negative for BV). To mitigate this, we applied synthetic minority oversampling technique (SMOTE) (Chawla et al., 2002). SMOTE generates synthetic data that over-samples the minority class (in this instance, observations with incident BV) and under-samples the majority class (i.e., those who remain negative for BV). Previous studies demonstrate that SMOTE effectively improves classification accuracy (Sun et al., 2012; Nakamura et al., 2013; Dai, 2015). After applying SMOTE, the samples of the training set increased from 168 to 209 observations in the meatal dataset (105 BV negative, 104 BV positive), and from 78 to 98 (50 BV negative, 48 BV positive) in the glans/coronal sulcus dataset. The oversampled training set was used for establishing the prediction models. The SMOTE data was generated using the Weka open source program (Frank et al., 2016).

### Statistical Analysis

The analysis proceeded in two steps: individual classification (Step 1) and ensemble learning (Step 2). Step 1. We applied three classification algorithms to estimate the predictive capacity of the baseline penile microbiome for incident BV: Random Forest (RF), Support Vector Machine (SVM), and K Nearest Neighbor (KNN). The individual classification algorithms were chosen to represent a diverse set of underlying assumptions, which produces a stronger ensemble (Zhou, 2012). Statnikov et al. have previously demonstrated SVM and RF to have superior classification performance on human microbiome data as compared to KNN and neural networks (Statnikov et al., 2013). Random Forest is a decision-tree technique that searches for the best variables that minimize training error when the decision is taken as the average of outcome on these regions. SVM identifies the decision boundary that maximizes the margin between two data classes (Cortes and Vladimir, 1995). In our analysis, we used a non-linear kernel for more consistent performance improvement (Zhou and Gallins, 2019). KNN differs from RF and SVM in that it uses a distance based metric. To avoid overfitting, we applied 10-fold cross-validation (repeated 1,000 times) for each classifier and for the voting algorithm (Ng, 1997). In the cross-validation, data is partitioned into training and testing datasets; the model developed in the training dataset is tested on an unknown subset (i.e., test dataset) to assess the ability to predict “new” data that was not used in estimating the model (Cawley and Talbot, 2010). To tune parameters of each model, we applied a grid search of possible values on the training set, then the test set was used to evaluate the performance of the model based on the selected optimal tuning parameters.

In Step 2, we used the three classifiers to predict the outcome of incident BV through majority voting. Majority voting counts the votes from the three classifiers and allocates a queried residue to the class that gains the majority votes. The motivation of ensemble learning is that by combining multiple classifiers, the prediction performance will benefit from their differing strengths to improve the prediction performance (Zhou, 2012). The performance of the classifiers was evaluated using area under the curve (AUC), accuracy, sensitivity, and specificity measures. We also included circumcision status in the prediction models, because of its known influence on penile bacterial community (Mehta et al., 2012; Liu et al., 2015) and female sex partner's BV status (Gray et al., 2009; Liu et al., 2015). To compare the prediction capability of the two datasets (meatal, glans-coronal sulcus-shaft), we then conducted a permutation *t*-test 1,000 times on the area under the curve (AUC) calculations for each comparison. Classification (Step 1) and estimation of accuracy (Step 2) were conducted in R using the *randomForest* (4.6–14), *e1071* (1.7–2), and *kknn* (1.3.1) packages. Important variables from each classifier were ranked: mean decrease in accuracy for RF, recursive feature elimination by classification performance for SVM (Guyon et al., 2002), and support criterion for KNN (Li et al., 2011). As there are currently no methods for identification of variable importance stemming from ensemble voting, we report the ensemble variable importance through averaging the ranks of the three classifiers. We also report the variable ranks for each classifier, and visualize overlaps with Venn diagrams (Bardou et al., 2014). We present the CLR-transformed values of the top 20 taxa with highest mean relative abundance heatmaps separately for meatal and glans/coronal sulcus, generated using *gplots*, with complete linkage clustering of the Euclidean distance. Differences in overall community composition by anatomic site and circumcision status are visualized with non-metric dimensional scaling (NMDS) plots based on Bray-Curtis similarity measure (conducted in Primer-e Clarke and Gorley, 2015). The R code necessary to enable the reproduction of the analysis and minimal data sets underlying the findings described and used to reach the conclusions of the manuscript are available as Supplementary Material.

## Results

Among 168 couples in which the woman did not have Nugent defined BV at baseline, the cumulative incidence of BV was 31.6%: 27.3% among women with circumcised male partner and 37.3% among women with uncircumcised male partner (*p* = 0.12). Among the 52 women with BV, 36 women had one episode of BV, 13 women had 2 episodes, and 3 women had 3 episodes. Over half (56%) of men were circumcised. Men commonly reported additional sex partners (21%), though only 3% of women reported having had a non-index sex partner in the past 6 months. Condom use at last sex was infrequent, reported by 16.7% of men and women. Characteristics of couples are shown in Table 1.

**Table 1:** Baseline characteristics of couples included in analyses.

**Characteristics**	**Couples with meatal samples**, ***N*** **=** **168**		**Couples with glans/coronal sulcus samples^∧^, *N* = 78**	
	**Men *n* (%)**	**Women *n* (%)**	**Men *n* (%)**	**Women *n* (%)**
Bacterial vaginosis (BV) status at follow-up				
Persistent negative		116 (69.0)		54 (69.2)
Incident		52 (31.0)		24 (30.8)
Nugent score at baseline				
0–3		142 (84.5)		69 (88.5)
4–6		26 (15.5)		9 (11.5)
Time in months to first incident BV, among women with Nugent score 0–3 at baseline				
1 month		13 (35.1)		4 (20.0)
6 months		16 (43.2)		10 (50.0)
12 months		8 (21.6)		6 (30.0)
Time in months to first incident BV, among women with Nugent score 4–6 at baseline				
1 month		10 (66.7)		4 (100)
6 months		4 (26.7)		
12 months		1 (6.7)		
Male partner circumcision status				
Circumcised Incident BV in• female partner	99 (58.9)	26 (26.3)	52 (66.7)	14 (26.9)
Uncircumcised Incident BV in• female partner	69 (41.1)	26 (37.3)	26 (33.3)	10 (38.5)
Median age, years (IQR)	27 (24–30)	23 (20–25)	27.5 (25–31)	24 (21–26)
Number of sex partners past 6 months				
1	132 (79.0)	163 (98.8)	56 (71.8)	76 (98.7)
2 or more	35 (21.0)	2 (1.2)	22 (28.2)	1 (1.3)
Missing	1	3		1
Condom used at last sex	28 (16.7)	28 (16.7)	14 (18.0)	14 (18.0)

∧ *The 78 couples in which glans/coronal sulcus sample is available from men are a subset of the 168 couples*.

### Community Composition

**Figure 1:**
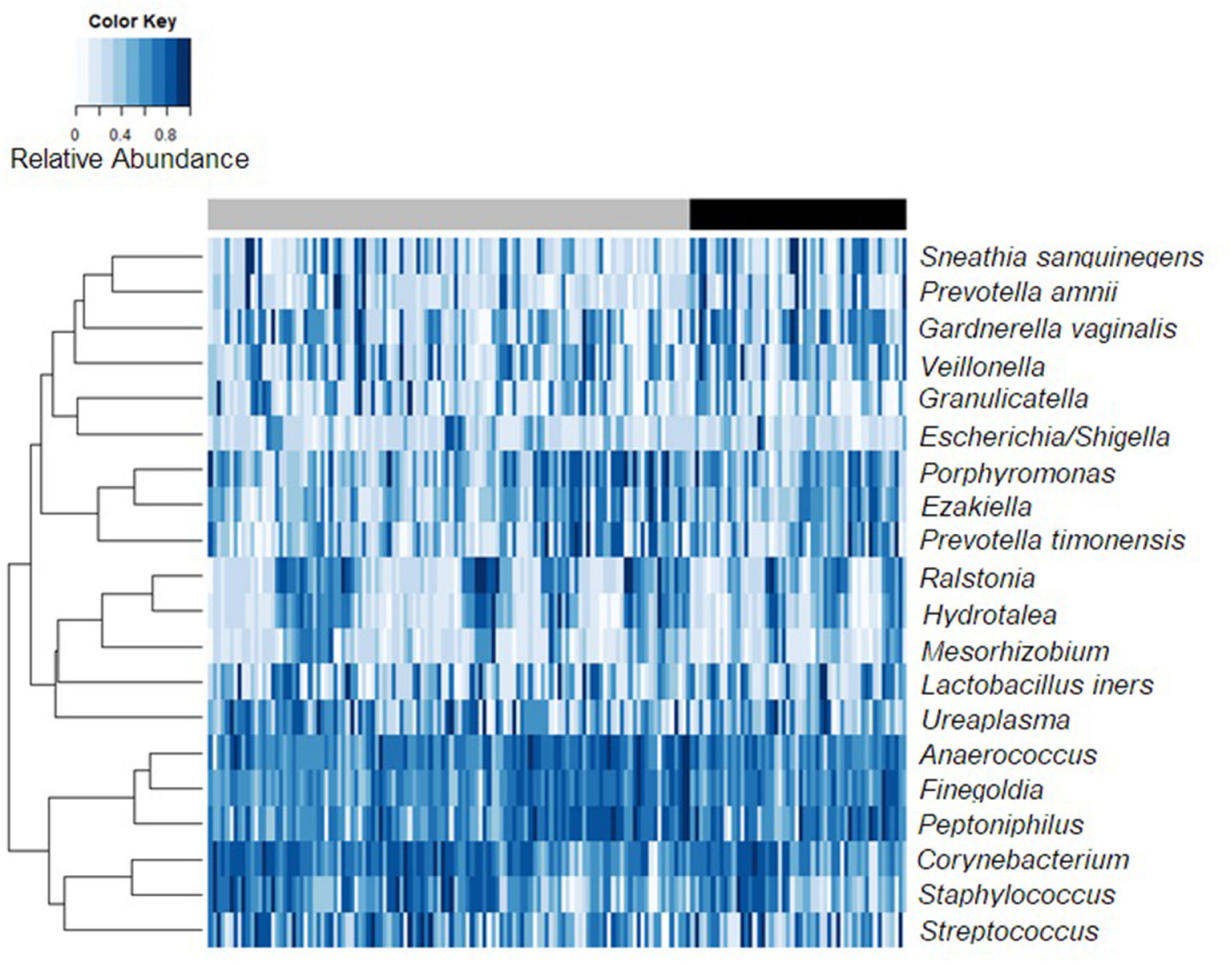
Bacterial relative abundance heatmap for 20 most abundant meatal taxa by incident Bacterial vaginosis status. Observations from 168 samples are sorted by female partner BV status. The top bar reflects observations where the female partner is persistently BV negative [gray] vs. those with incident BV [black].

The relative abundances of the 20 most abundant meatal taxa for each observation are shown in Figure 1, sorted by incidence of BV among female partners (heatmap of glans/coronal sulcus shown in Supplemental Figure 1). Presence and mean relative abundance of the top 20 meatal taxa are reported at the aggregate level (Table 2; glans/coronal sulcus in Supplemental Table 1). The taxa with the highest relative abundances were similar for meatal and glans/coronal sulcus samples (e.g., *Corynebacterium, Streptococcus, Anaerococcus, Finegoldia*), but the overall composition was distinct (Supplemental Figure 2). The taxa with highest prevalence and relative abundance differed by circumcision status (Supplemental Tables 2, 3, Supplemental Figures 3, 4).

**Table 2:** Presence and mean relative abundance of 20 most abundant meatal taxa by incident Bacterial vaginosis (BV) status.

	**Presence**		**Mean relative abundance, % (SD)**	
	**Female partner remained BV negative, *N* = 116 *n* (%)**	**Female partner had incident BV, *N* = 52 *n* (%)**	**Female partner remained BV negative, *N* = 54**	**Female partner had incident BV, *N* = 24**
*Corynebacterium*	115 (99)	50 (96)	18.3 (21.3)	15.7 (17.9)
*Streptococcus*	94 (81)	38 (73)	8.89 (16.7)	11.0 (22.2)
*Anaerococcus*	114 (98)	51 (98)	9.69 (9.82)	7.97 (7.86)
*Finegoldia*	115 (99)	51 (98)	8.27 (11.9)	7.06 (9.84)
*Lactobacillus iners*	67 (58)	34 (65)	6.28 (15.2)	8.62 (19.6)
*Peptoniphilus*	107 (92)	47 (90)	5.40 (7.05)	5.86 (8.10)
*Staphylococcus*	105 (91)	44 (85)	5.96 (10.3)	4.66 (8.54)
*Sneathia sanguinegens*	44 (38)	28 (54)	4.04 (11.1)	6.41 (12.4)
*Ralstonia*	59 (51)	25 (48)	4.62 (12.2)	2.60 (8.45)
*Ezakiella*	80 (69)	43 (83)	3.13 (9.20)	3.02 (7.01)
*Veillonella*	64 (55)	33 (63)	2.54 (5.94)	4.04 (8.35)
*Gardnerella vaginalis*	56 (48)	34 (65)	1.86 (4.64)	3.34 (5.65)
*Prevotella timonensis*	62 (53)	33 (63)	1.60 (3.89)	2.01 (5.35)
*Porphyromonas*	78 (67)	33 (63)	1.95 (4.20)	1.21 (3.07)
*Granulicatella*	43 (37)	19 (37)	1.49 (6.09)	0.56 (1.66)
*Prevotella amnii*	20 (17)	16 (31)	1.05 (4.04)	1.17 (2.89)
*Ureaplasma*	75 (64)	29 (56)	1.15 (2.86)	0.93 (3.42)
*Hydrotalea*	56 (48)	22 (42)	1.30 (4.40)	0.52 (1.22)
*Mesorhizobium*	36 (31)	15 (29)	0.79 (4.17)	0.47 (2.19)
*Escherichia Shigella*	24 (21)	12 (23)	0.51 (3.47)	0.99 (6.45)

*SD, Standard Deviation; BV, Bacterial vaginosis*.

### Classification Performance

The three classifiers performed comparably to each other in overall accuracy of predicting BV but varied substantially in how this was achieved, either through increased sensitivity or specificity. In the meatal dataset (Table 3), KNN had the greatest accuracy predicting BV (77.1%) compared to RF (73.3%) and SVM (74.0%), with superior sensitivity (95.2%), but with resultant tradeoff in specificity (60.9 vs. 72.4% for SVM and 77.2% for RF). The pattern was similar for the glans/coronal sulcus samples (Supplemental Table 4). In the meatal dataset, although of small difference, voting had greater accuracy compared to the individual classifiers, but this was achieved through a balance of optimizing sensitivity and specificity. For example, though RF had 77.2% specificity in the meatal samples, the sensitivity was 69.0%. When combined with the other classifiers, the overall specificity was 74.6% and sensitivity was 80.7%.

**Table 3:** Classification performance for prediction of incident Bacterial vaginosis in women by male partner's meatal microbiome.

	**Random forest**	**Support vector machine**	**K nearest neighbor**	**Voting**
Accuracy	0.733	0.740	0.771	0.775
Specificity	0.772	0.724	0.609	0.746
Sensitivity	0.690	0.757	0.952	0.807
Area under the curve (AUC)	0.790	0.827	0.889	0.888

**Figure 2:**
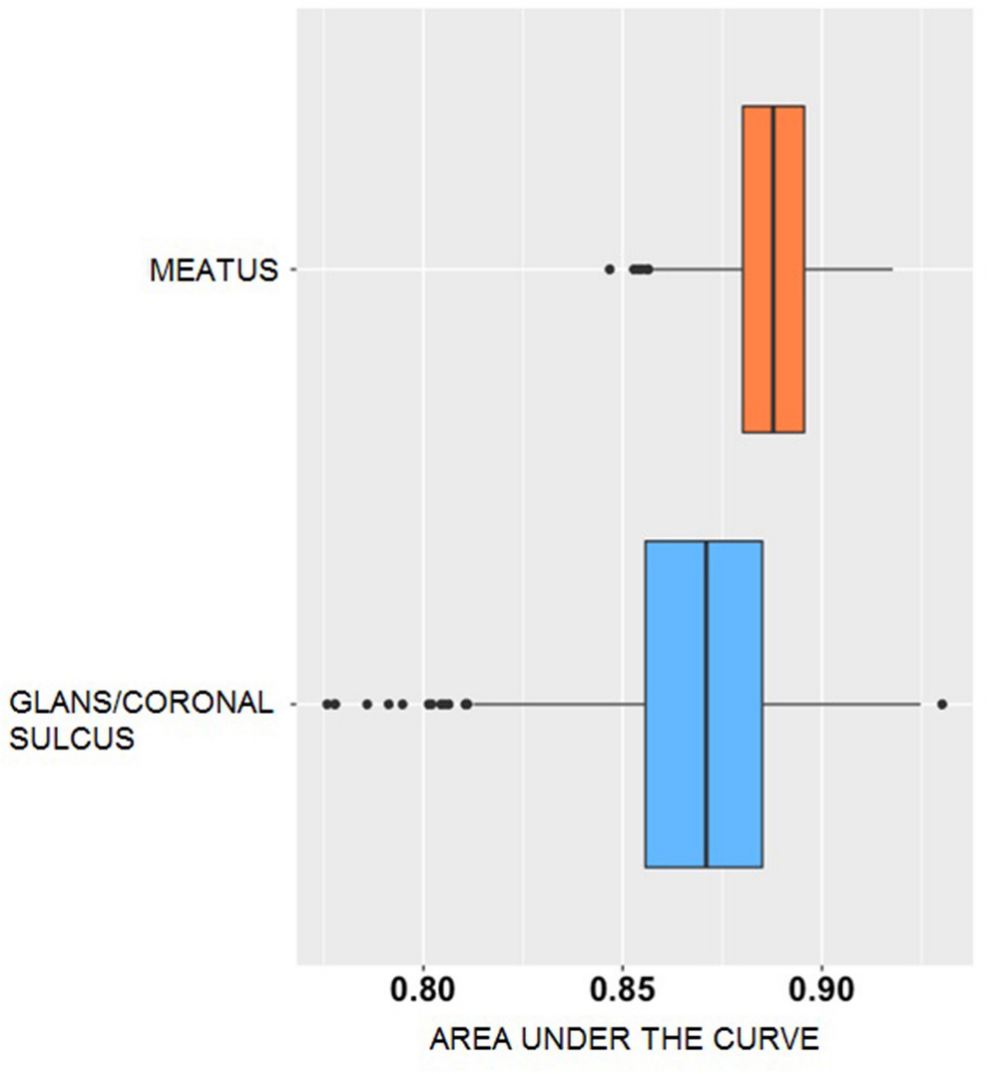
AUC distribution generated from voting classification of incident Bacterial vaginosis for penile microbiomes. The x-axis represents the area under the curve (AUC) of the predictive accuracy. The y-axis indicates the bacterial dataset, meatal (orange) or glans/coronal sulcus (blue). The box plots show the median (centerline), upper and lower quartiles (box shoulders), and outliers (black dots). The results are based on 1,000 simulations.

The area under the curve (AUC) difference was 0.02 (88.8% meatal vs. 86.9% glans/coronal sulcus; permutation test *p* < 0.001; 1,000 simulations), indicating that although classification accuracy is similar for meatal and glans/coronal sulcus samples, meatal AUC has a slight performance advantage. However, although prediction from these two anatomic sites produced similar accuracy and AUC, the AUC of glans/coronal sulcus is more widely spread with several outliers (Figure 2). The AUC of meatal bacteria has smaller variance compared to glans/coronal sulcus, and thus more stable prediction performance, though this may have been due to a larger number of meatal observations.

### Key Penile Taxa Involved in the Prediction of Incident Bacterial Vaginosis

**Figure 3:**
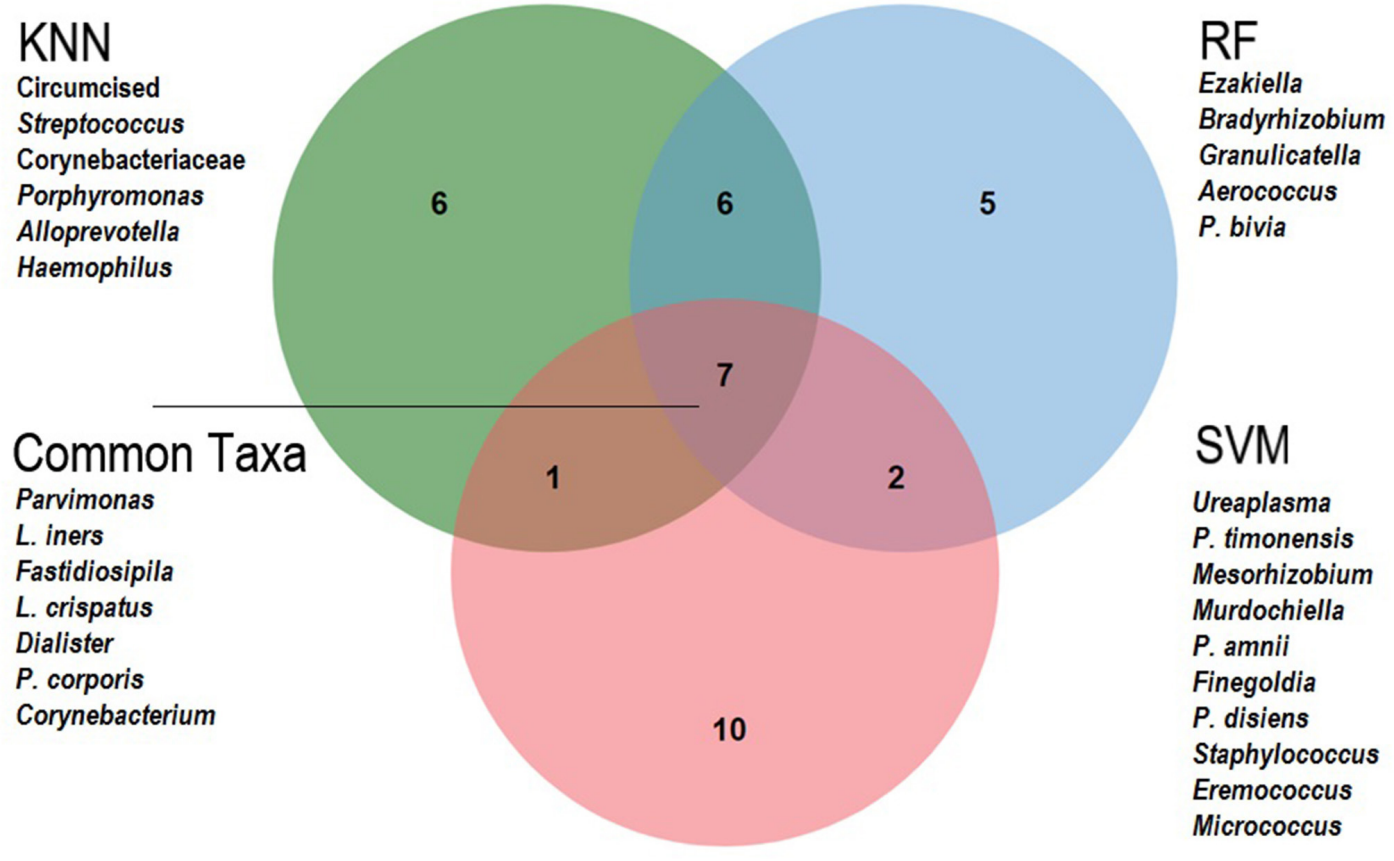
Venn Diagram of 20 top-ranked meatal taxa predicting Bacterial vaginosis, by machine learning classifier. This Venn diagram shows the overlap of the top 20 important variables from each classifier: KNN, K Nearest Neighbor; RF, Random Forest; SVM, Support Vector Machine. The unique variables are listed for each classifier and the common taxa across all three are listed as indicated.

Variable ranking was applied to determine which taxa had most impact in the prediction models. Based on average ranks across the three classifiers (Table 4), the 10 most important meatal taxa in predicting incident BV were (in descending order of rank importance): *Parvimonas, Lactobacillus iners, Fastidiosipila, Negativicoccus, L. crispatus, Dialister, Sneathia sanguinegens, Gardnerella vaginalis, Prevotella corporis*, and *Corynebacterium*. When comparing the top 20 most important taxa for predicting BV from each classifier (Figure 3), the seven taxa shared across all three classifiers were *Parvimonas, L. iners, Fastidiosipila, L. crispatus, Dialister, P. corporis*, and *Corynebacterium*. Circumcision status was ranked relatively low (17th) across the classifiers for meatal taxa, and was not in the top 20 for glans/coronal sulcus taxa. Based on average ranks across the three classifiers, none of the 10 most important glans/coronal sulcus taxa in predicting incident BV overlapped with the top 10 meatal taxa predicting incident BV (Supplemental Table 5). When comparing the top 20 most important taxa for predicting BV from each classifier for the glans/coronal sulcus (Supplemental Figure 5), five taxa shared across the three classifiers were *Enhydrobacter, Brevibacterium, P. bivia, Staphylococcus, and P. buccalis*.

**Table 4:** Variable importance ranking by classifier method and for voting from meatal samples: top 20 Taxa by voting.

**Variable**	**K-nearest neighbor rank**	**Random forest rank**	**Support vector machine rank**	**Voting rank**
*Parvimonas*	3	4	4	1
*Lactobacillus iners*	6	1	13	2
*Fastidiosipila*	5	12	11	3
*Negativicoccus*	1	2	27	4
*Lactobacillus crispatus*	10	20	7	5
*Dialister*	16	5	19	6
*Sneathia sanguinegens*	11	7	22	7
*Gardnerella vaginalis*	8	8	26	8
*Prevotella corporis*	18	15	9	9
*Corynebacterium*	17	16	16	10
*Sneathia amnii*	4	9	42	11
*Chryseobacterium*	33	10	14	12
*Acinetobacter*	7	6	45	13
*Escherichia shigella*	15	45	2	14
*Gemella*	40	3	20	15
*Prevotella timonensis*	30	28	5	16
Circumcised (vs. uncircumcised)	9	27	32	17
*Peptostreptococcus*	2	18	49	18
*Ezakiella*	21	11	43	19
*Alloprevotella*	19	23	36	20

*This table shows the 20 top-ranked taxa by voting, and their variable importance ranking according to each of the other classifiers. The voting importance ranking is determined by averaging the rank of across the three classifiers. We use conditional formatting (Excel) to facilitate reading ranks across the three classifiers, whereby red represents taxa ranked with higher importance and blue represents taxa that are ranked with lower importance*.

### Sensitivity Analyses Excluding Observations in Which the Female Partner had Intermediate Nugent Score at Baseline

Because intermediate Nugent scores (4–6) may represent misclassified BV or BV that is in process of developing, we conducted sensitivity analyses in which we excluded observations in which the female partner had intermediate Nugent score at baseline. In the meatal dataset (*n* = 142; Supplemental Table 6), the AUC was 96.9% and was 94.7% in the glans/coronal sulcus dataset (*n* = 69; Supplemental Table 7). In both datasets, the increase in AUC was achieved through gain in specificity in all three classifiers. In both datasets, SVM had the greatest accuracy of the three classifiers in predicting BV, having sensitivity, specificity, accuracy, and AUC similar to that of voting. Variable importance ranking shows that many of the top 20 taxa by voting are similar between the main analysis and the sensitivity analyses for both meatal (Supplemental Table 8) and glans/coronal sulcus samples (Supplemental Table 9), which supports the stability of the findings. In the meatal samples, the six taxa shared across all three classifiers were *Veillonella, Negativicoccus, S. amnii, Brevibacterium, G. vaginalis*, and *Parvimonas* and *Fastidiosipila*, the last two also being in the top taxa shared across taxa in main analyses. In the glans/coronal sulcus samples, the six taxa shared across all three classifiers were *Ruminococcaceae ucg014, Rothia, Kocuria, Peptostreptococcus*, Peptostreptococcaceae, and *Brevibacterium*, with only *Brevibacterium* being in the top taxa shared across taxa in the main analyses.

## Discussion

We examined the questions, (1) what is the predictive capacity of the penile microbiome for incident BV in female sex partners, and (2) does this differ by penile sampling site. Using assimilation of multiple machine learning classifiers, this study had four main findings: (1) The penile microbiome accurately predicts incident BV in the female sex partner; (2) Prediction accuracy of incident BV in female sex partners was similar for microbiota from the meatus and from glans/coronal sulcus, though results from meatal samples had more stable performance; (3) Several meatal bacteria that were most important in predicting incident BV overlapped with vaginal bacteria commonly associated with BV, which intuitively explains the high predictive accuracy for incident BV in female partners, whereas this was less apparent for bacteria from the glans/coronal sulcus; and (4) Circumcision status had low variable importance in the predictions of incident BV in female partners.

Both penile sites had similar and high predictive accuracy for incident BV (77.5% meatal, 76.8% glans/coronal sulcus). BV-associated bacteria were recovered from both the meatal and glans/coronal sulcus samples, though with greater relative abundances in the meatus. This is in keeping with previous studies that have identified BV-associated bacteria in semen, urethral, and urine samples (Nelson et al., 2012; Mandar et al., 2015; Zozaya et al., 2016). While circumcision of the male partner has been associated with reduced BV among female sex partners, in our classifiers it had low variable importance ranking in relation to incident BV in both the meatal and glans/coronal sulcus data, except in KNN classification of meatal data. This makes sense because the likely mechanism by which circumcision protects against BV is through reduction of the anaerobic bacteria within the meatus and glans/coronal sulcus, and thus circumcision status itself is not an important predictor, but rather the penile microbial composition itself. One theory is that recovery of penile bacteria that in the vagina are associated with BV, may simply represent recent or frequent sex and it is the recent or frequent sex that explains the association with BV in the female partner; however, this explanation cannot account for the differential association between circumcision status and BV observed in our study and others (Gray et al., 2009; Liu et al., 2015). An alternative explanation is that the association between circumcision status and BV is due to the higher penile bacterial load for uncircumcised men, rather than the composition itself. However, the cross-sectional study by Liu et al. found associations between penile microbiome composition and female partner BV status considering both bacterial density and proportional abundance (Liu et al., 2013). Adding to the epidemiologic and microbiologic evidence that implicates the penile microbiome in BV, our data show a clear temporal association between penile microbial composition and the subsequent development of disease. The results provide empiric justification for evaluating the effect of male partner treatment on female partner risk of BV or recurrence. Our results show that any potential treatment—antibiotic or live biotherapeutic—will need to be effective in reducing or altering the bacteria at both the meatus and the glans and coronal sulcus. In a pilot study of 22 couples in which women had symptomatic BV, male partners treated with oral metronidazole 400 mg twice daily and 2% clindamycin cream topically for 7 days exhibited a reduction in the prevalence and abundance of BV-associated bacteria in the glans/coronal sulcus 8 days following treatment (Plummer et al., 2018), though this was not sustained at 28 days. Plummer et al. report this was not related to re-emergence of BV-associated bacteria among female sex partners and hypothesize that return to baseline penile microbial composition may be due to BV-associated taxa persisting in the penile skin, urethra, or prostate, or re-introduction of BV-associated bacteria from oral sex or the gastrointestinal tract. Moreover, Plummer et al. did not have urethral data for all males and the effect of antimicrobial treatment on the urethral microbiome was not explored in detail. Given the variability across couples in their study, larger studies of longer duration are needed to understand the potential implications of antimicrobial treatment on the penile microbiome and female partner recurrence of BV.

Comparing the top 20 important taxa across classifiers, seven meatal taxa were shared across all three: *Parvimonas, Fastidiosipila, Negativicoccus, P. corporis*, and *Corynebacterium*. Vaginal *Parvimonas, L. iners, L. crispatus, Fastidiosipila*, and *Prevotella* (at the genus level and multiple species) have previously been linked to BV and diverse vaginal community (Fredricks, 2011; van de Wijgert et al., 2014). A recent study of South African men identified seven community types of penile microbiota; *Negativicoccus* was enriched in a diverse community type that also had penile enrichment with BV-associated taxa (e.g., *Dialister, Sneathia, Gardnerella*), and a higher prevalence of BV among female partners (Oynwera et al., 2020). Additionally, *Negativicoccus* bacterial load and presence is significantly reduced in men undergoing circumcision (Liu et al., 2013). These penile taxa may be contributing directly to BV through transmission to the vaginal microbiome, or they may be contributing to overall diversity or perturbation of the vaginal microbiome. While vaginal *Corynebacterium* is not associated with BV, penile *Corynebacterium* is more abundant among circumcised men (Liu et al., 2013) and may represent a suite of differences in microbiome composition and function between circumcised and uncircumcised men. Vaginal *L. iners* and *L. crispatus* are generally inversely associated with BV; here as the predictive algorithms do not produce coefficients or directionality, it is unclear the nature of the association of penile *L. crispatus* with incident BV. Future studies should evaluate the longitudinally interacting or mediating effects of penile microbiome on the vaginal microbiome in predicting BV to help uncover the underlying mechanism(s). Addressing the methodologic gap in rigorously tested methods for longitudinal, microbiome-to-microbiome paired analysis could improve the accuracy of the prediction, further elucidate taxa of importance (which is informative to therapeutic and preventive strategies), and provide understanding of mechanisms by which the penile microbiome contributes to BV. Such analytic methods would advance studies of paired, longitudinal microbiome composition broadly (e.g., mother-infant pairs, individual-household, multiple anatomic sites within individuals over time).

The prediction accuracies from the meatal and glans/coronal sulcus samples were similar, but with different variable importance rankings, which likely stems from the differing microbiome compositions at these sites. This is important for future studies examining the penile microbiome in association with BV or other outcomes associated with non-optimal vaginal microbiome (e.g., sexually transmitted infections, HIV, adverse pregnancy outcomes). For both circumcised and uncircumcised men, measuring one penile site may be sufficient, with the meatal site potentially having superiority due to greater reliability and consistency in generation of biologically valid findings. Sampling and testing only one penile site reduces costs and promotes standardization for comparability across studies of circumcised and uncircumcised men. However, future studies of penile microbiome and outcomes in men and their female partners should replicate this investigation. While the observations in the glans/coronal sulcus dataset were a representative subset of the observations in the meatal dataset, the meatal dataset was more than double the sample size of the glans/coronal sulcus sample size. The larger sample size for meatal observations would have contributed to the improved precision in the AUC estimates. However, the variance and outliers in the AUC of the glans/coronal sulcus estimates may have been driven by variable selection with less consistency across classifiers; variability may also have been driven by high importance ranking of several taxa with low relative abundance and without previously demonstrated association with BV in women (e.g., *Enhydrobacter, Brevibacterium*, Ruminococcaceae*, Campylobacter, Rothia)*. However, it should be noted that *Enhydrobacter* and *Brevibacterium*, both with high variable importance ranking, have been detected in the semen or penile microbiome with relatively high prevalence and abundance (Liu et al., 2013; Plummer et al., 2018). Among Black South African men, penile *Campylobacter* has been observed to be differentially abundant by men's high-risk human papilloma virus status and HIV-infected men had greater relative abundance of *Ruminococcus* (Oynwera et al., 2020). In a small sample of men who have sex with men, *Rothia* was among the most prevalent bacteria recovered from semen and was detected in 86% of HIV-uninfected men and compared to 48% of HIV-infected men (Liu et al., 2014) and *Rothia* increases among men undergoing medical male circumcision (Liu et al., 2013).

Some studies have shown daily and weekly temporal fluctuation in the vaginal microbiome (Fredricks, 2011), while others have shown temporally durable persistence of dominant taxa (Gajer et al., 2012). Similar studies have not been done of the penile microbiome. In our study, baseline penile microbiota accurately predicted BV incidence up to 1 year later in women who did not have BV at baseline, with more than half of incident infections observed at 6- to 12-months after penile microbiome assessment. This suggests that the penile microbiome is relatively stable, or that the penile microbiota contribution to BV risk accumulates over time. As noted above, with methodologic advances, subsequent analyses could help elucidate this by evaluating change over time in the BV-associated penile taxa and association with changes over time in the vaginal taxa.

We conducted three machine learning approaches to classify BV, and applied voting to summarize across these classifications. Each classifier achieved similar predictive accuracy, but with varying trade-offs in sensitivity and specificity, which reflects the differing underlying properties of the classifiers. Once voting was applied, accuracy and AUC were maximized, with a balance of high sensitivity and specificity, drawing on the differing strengths of the individual classifiers. While there was substantial overlap in important taxa across the three classifiers, differences between the rankings could be due to taxa that have similar function, and studies of bacterial function will help elucidate this. The AUC was superior (96.7 meatal, 94.7 glans/coronal sulcus) in sensitivity analyses that excluded observations in which the female partner had an intermediate Nugent score at baseline. The basis of performance gain in specificity, especially for the SVM classifier is unclear. However, the sensitivity analysis strengthens the robustness of findings in that penile microbiome composition was highly predictive of incident BV in female sex partners who had Nugent score 0–3 at the time the penile microbiome was measured, and the top 20 meatal and glans/coronal sulcus taxa were largely similar to main analyses. Our findings exemplify the advantage of ensemble methods to improve prediction by reducing error through averaging of the independent classifiers. Prior to classification, we applied SMOTE to address the imbalanced outcome distribution (BV incidence) in our sample, as prediction within the original dataset was weighted heavily to specificity in both the meatal (Supplemental Table 10) and glans/coronal sulcus (Supplemental Table 11) datasets. Brooks et al. evaluated race/ethnicity differences in the gut microbiome using data from the Human Microbiome Project, where they also report SMOTE sampled data generated prediction accuracy that was superior to no sampling (Brooks et al., 2018). While relatively new to microbiome analyses, SMOTE is a common approach to address imbalanced datasets in machine learning predictive analyses.

## Limitations

A limitation inherent in amplicon sequencing is annotation of bacteria. While a standardized and replicable approach was applied (Holm et al., 2019), this algorithm has not been optimized for the penile microbiome. Bacteria that are not represented in the reference database may be misclassified by grouping with a sequence that is most similar. Future studies employing long-read amplicon sequencing will also improve annotation accuracy. We used the baseline penile microbiome measure rather than the most temporally proximal penile microbiome measurement due to variably missing follow up visits. This reflects the challenges of enrolling and following a cohort of community-recruited couples who are not receiving an intervention. We could not examine predictive accuracy as a function of time since baseline, given the small number of incident infections at any single time point. Despite this, the baseline penile microbiome demonstrated high predictive accuracy for incident BV—including 56% of incidents occurring 6–12 months after baseline.

## Conclusions

The results of this study demonstrate the meatus and glans/coronal sulcus are reservoirs for BV-associated bacteria for both circumcised and uncircumcised men, and that bacteria from these penile sites have high predictive accuracy for incident BV occurring up to 6–12 months in the future in female sex partners. Longitudinal studies examining the role of the penile microbiome in vaginal microbiome outcomes in women, and randomized trials examining the potential effect of microbiome-altering treatments on the penile microbiome and in relation to preventing BV recurrence are warranted.

## Data Availability Statement

The datasets presented in this study can be found in online repositories. The names of the repository/repositories and accession number(s) can be found in the article/Supplementary Material.

## Ethics Statement

The studies involving human participants were reviewed and approved by the Ethical Review Committee of Maseno University (Kisumu, Kenya; MSU/DRPC/MUERC/00054/13; January 13, 2014), and the Institutional Review Board of the University of Illinois at Chicago (USA; 2013-0511; February 12, 2014). Written informed consent from the participants' legal guardian/next of kin was not required to participate in this study in accordance with the national legislation and the institutional requirements.

## Author Contributions

SM: obtained funding, study conceptualization and design, and drafted manuscript. DZ: design and implementation of statistical analysis approach (specifically, selection of machine learning algorithms and ensemble voting approach and variable importance ranking), visualization, critical review, and revision of manuscript. SG: development and oversight of protocols for amplicon sequencing, microbiologic analyses and interpretation, critical review, and revision of manuscript. WA: development, implementation and oversight of laboratory protocols in Kenya, acquisition of data, microbiologic analyses and interpretation, critical review, and revision of manuscript. FO: study oversight and management to ensure integrity to protocols, critical review, and revision of manuscript. RB: design and interpretation of statistical analysis approach (specifically, implementation of synthetic minority oversampling technique and optimizing parameters for prediction), critical review, and revision of manuscript. DB: conceptualization and design of statistical analysis approach (specifically, evaluating overall approach and results), critical review, and revision of manuscript. RCB: study oversight and management to ensure integrity to protocols, critical review, and revision of manuscript. All authors: contributed to the article and approved the submitted version.

## Conflict of Interest

The authors declare that the research was conducted in the absence of any commercial or financial relationships that could be construed as a potential conflict of interest.

## Supplementary Material

Supplemental Figure 1 Bacterial relative abundance heatmap for 20 most abundant glans/coronal sulcus taxa by incident Bacterial vaginosis status. Observations from 78 samples are sorted by female partner BV status [persistently negative (gray) vs. incident (black)].

Supplemental Figure 2 Non-metric dimensional scaling plot of meatal and glans/coronal sulcus bacterial communities using bray curtis similarity. This figure shows the relative similarity of the bacterial community of meatal samples (blue triangles ▲) to that of glans/coronal sulcus (red triangles ▼). The communities overlap, though the glans/coronal sulcus samples generally cluster more closely with each other.

Supplemental Figure 3 Non-metric dimensional scaling plot of meatal bacterial communities by circumcision status. This figure shows the relative similarity of the meatal bacterial community of circumcised samples (red triangles ▼) to that of uncircumcised samples (blue triangles ▲). The communities overlap, though there is generally more similarity of the circumcised samples to each other circumcised samples, and of uncircumcised samples to other uncircumcised samples.

Supplemental Figure 4 Non-metric dimensional scaling plot of meatal and glans/coronal sulcus bacterial communities. This figure shows the relative similarity of the glans/coronal sulcus bacterial community of circumcised samples (red triangles ▼) to that of uncircumcised samples (blue triangles ▲). There is little overlap in the communities.

Supplemental Figure 5 Venn Diagram of 20 top-ranked glans/coronal sulcus taxa predicting Bacterial vaginosis, by machine learning classifier. This Venn diagram shows the overlap of the top 20 important variables from each classifier: KNN, K Nearest Neighbor; RF, Random Forest; and SVM, Support Vector Machine. The unique variables are listed for each classifier and the common taxa across all three are listed as indicated.
