# Supplementary figures and images for: The Microbiome Composition of a Man's Penis Predicts Incident Bacterial Vaginosis in His Female Sex Partner With High Accuracy
Source: Front Cell Infect Microbiol. 2020 Aug 4;10:433. doi: 10.3389/fcimb.2020.00433 (PMC7438843; doi:10.3389/fcimb.2020.00433)

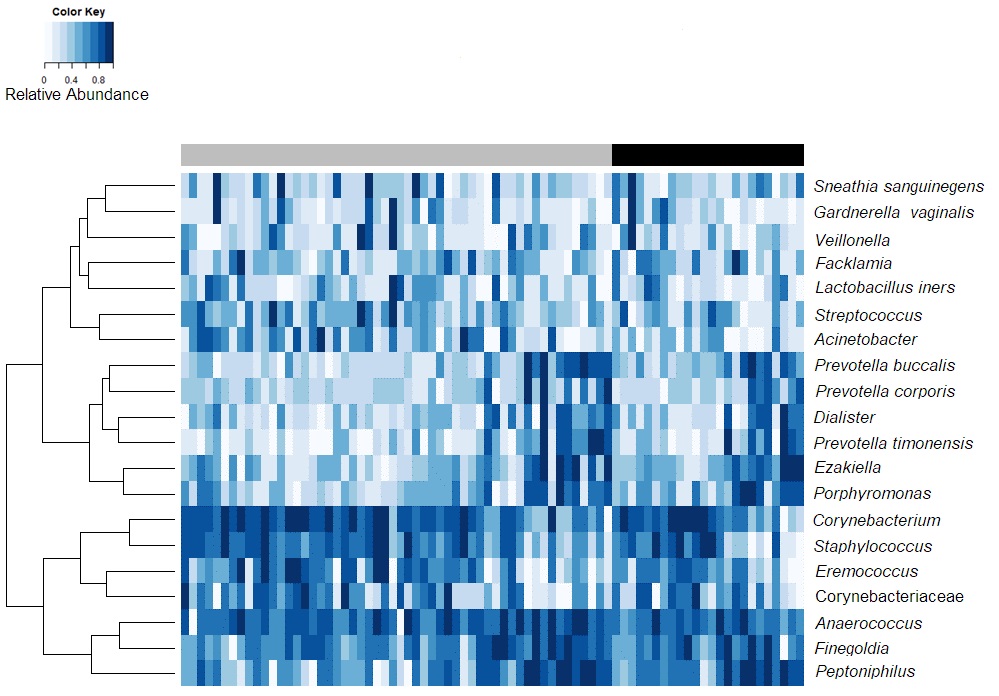

Supplement: Supplemental Figure 1 — Bacterial relative abundance heatmap for 20 most abundant glans/coronal sulcus taxa by incident Bacterial vaginosis status. Observations from 78 samples are sorted by female partner BV status [persistently negative (gray) vs. incident (black)]. [file Image_1.JPEG]

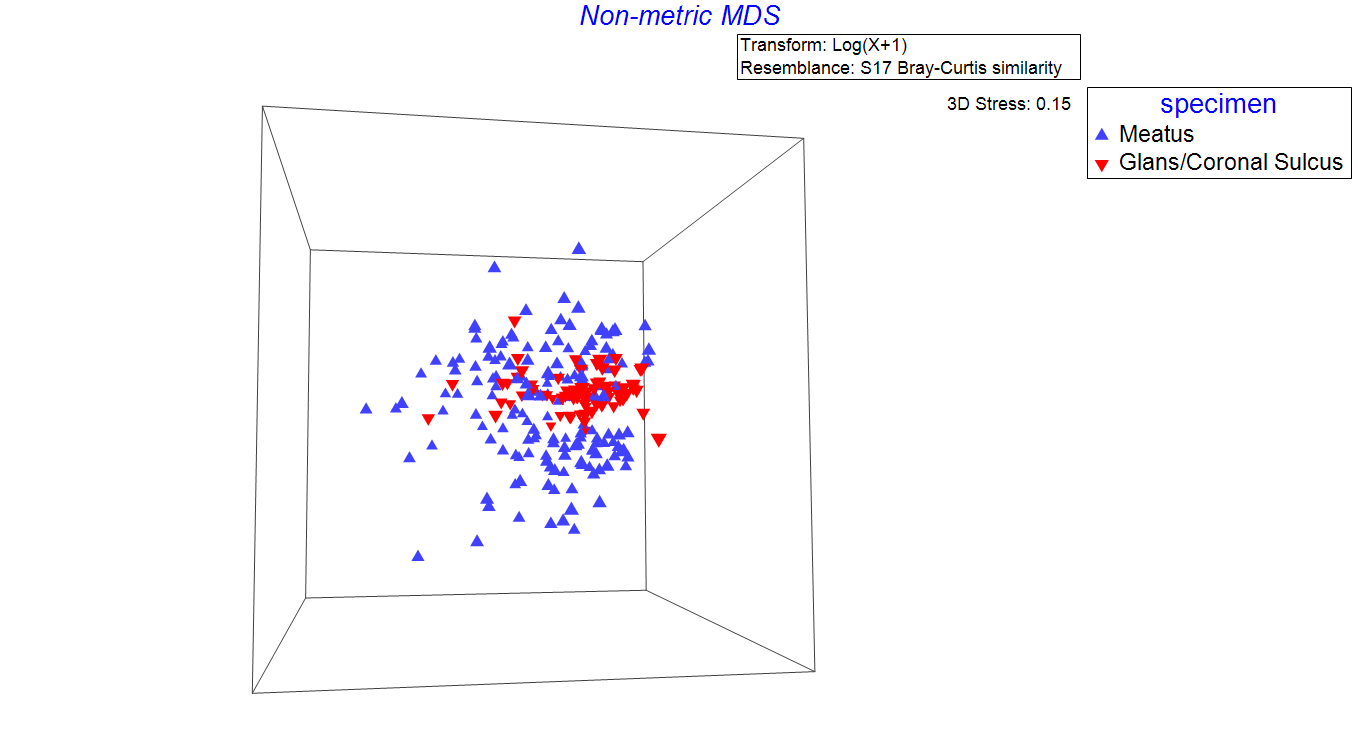

Supplement: Supplemental Figure 2 — Non-metric dimensional scaling plot of meatal and glans/coronal sulcus bacterial communities using bray curtis similarity. This figure shows the relative similarity of the bacterial community of meatal samples (blue triangles ▲) to that of glans/coronal sulcus (red triangles ▼). The communities overlap, though the glans/coronal sulcus samples generally cluster more closely with each other. [file Image_2.PNG]

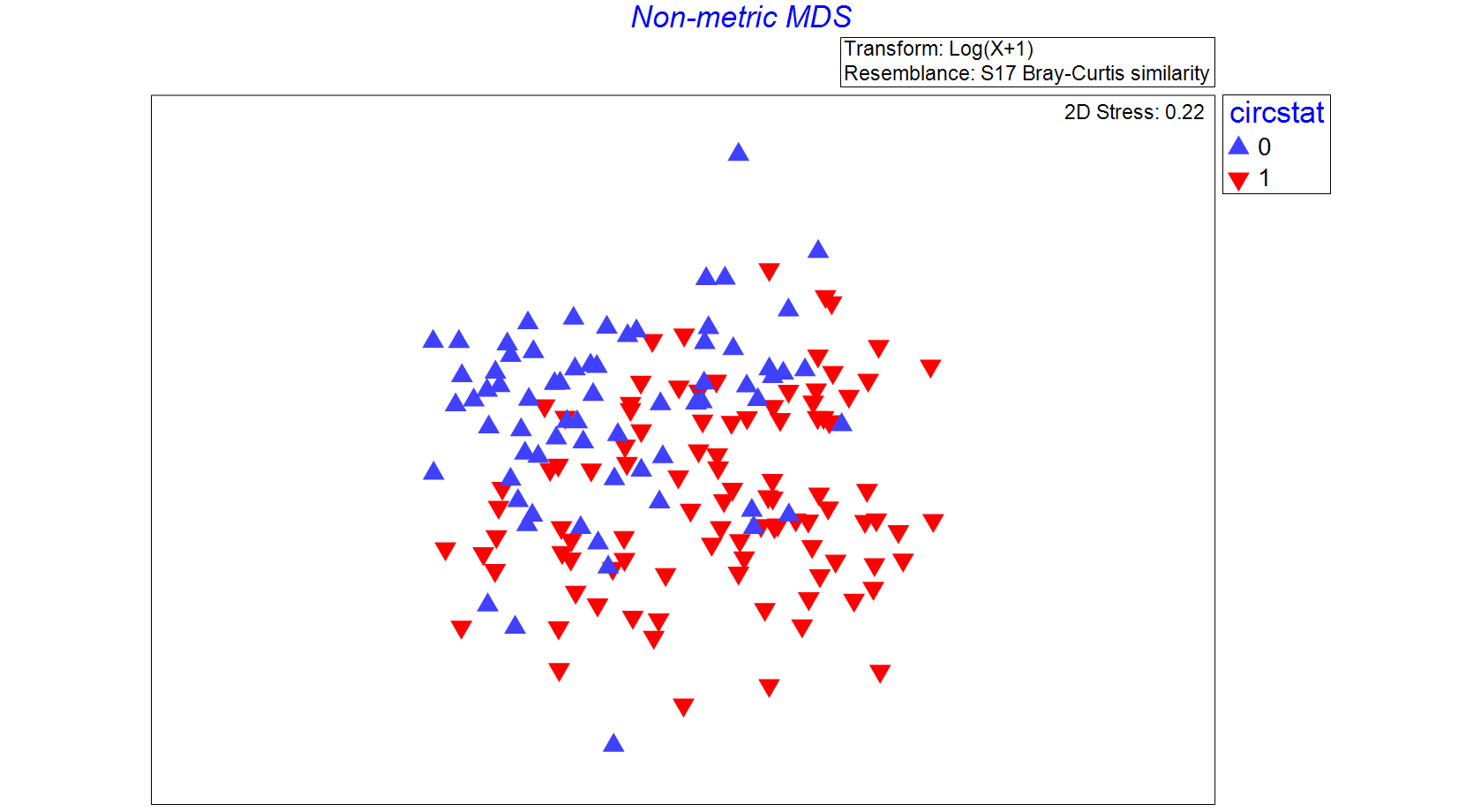

Supplement: Supplemental Figure 3 — Non-metric dimensional scaling plot of meatal bacterial communities by circumcision status. This figure shows the relative similarity of the meatal bacterial community of circumcised samples (red triangles ▼) to that of uncircumcised samples (blue triangles ▲). The communities overlap, though there is generally more similarity of the circumcised samples to each other circumcised samples, and of uncircumcised samples to other uncircumcised samples. [file Image_3.PNG]

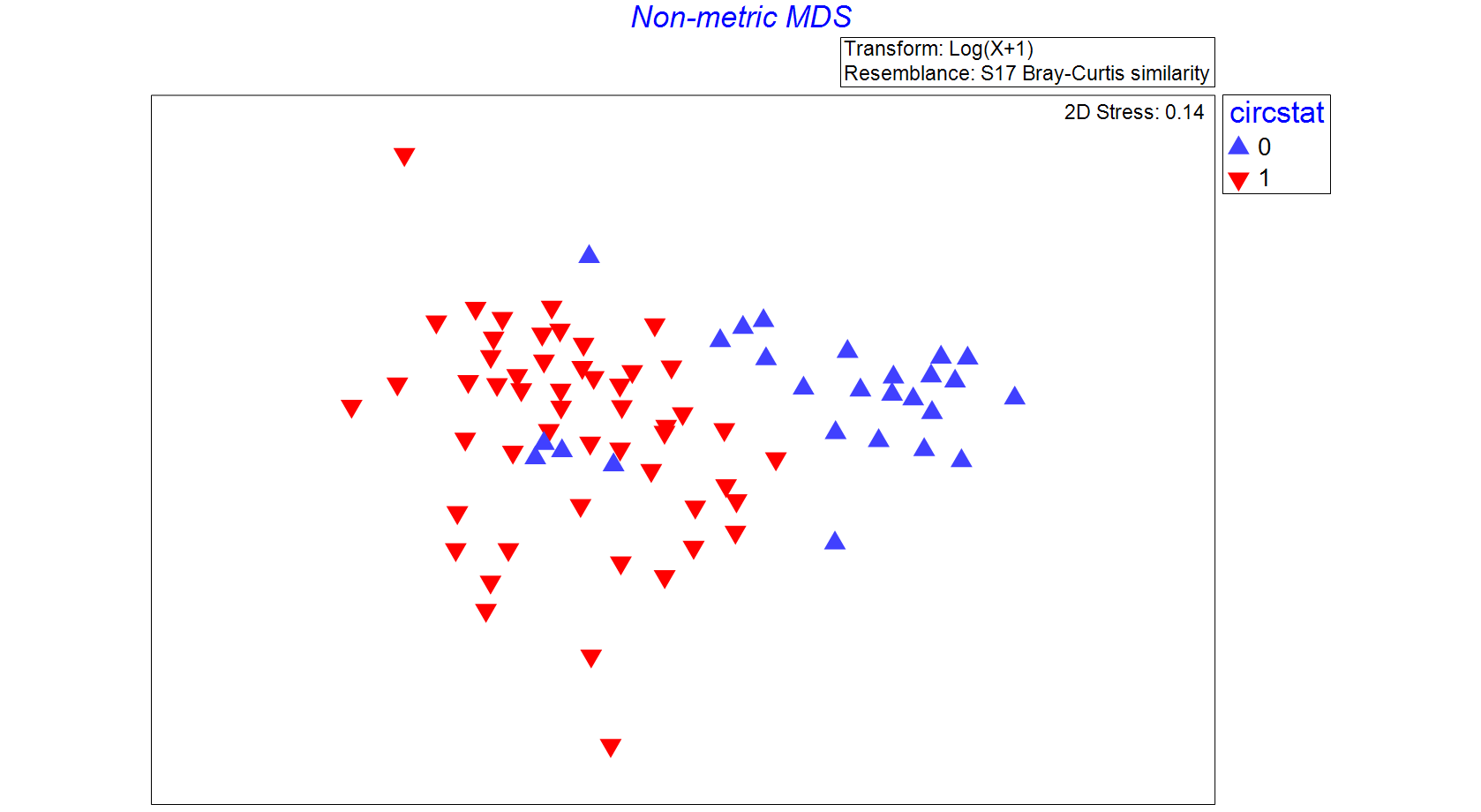

Supplement: Supplemental Figure 4 — Non-metric dimensional scaling plot of meatal and glans/coronal sulcus bacterial communities. This figure shows the relative similarity of the glans/coronal sulcus bacterial community of circumcised samples (red triangles ▼) to that of uncircumcised samples (blue triangles ▲). There is little overlap in the communities. [file Image_4.PNG]

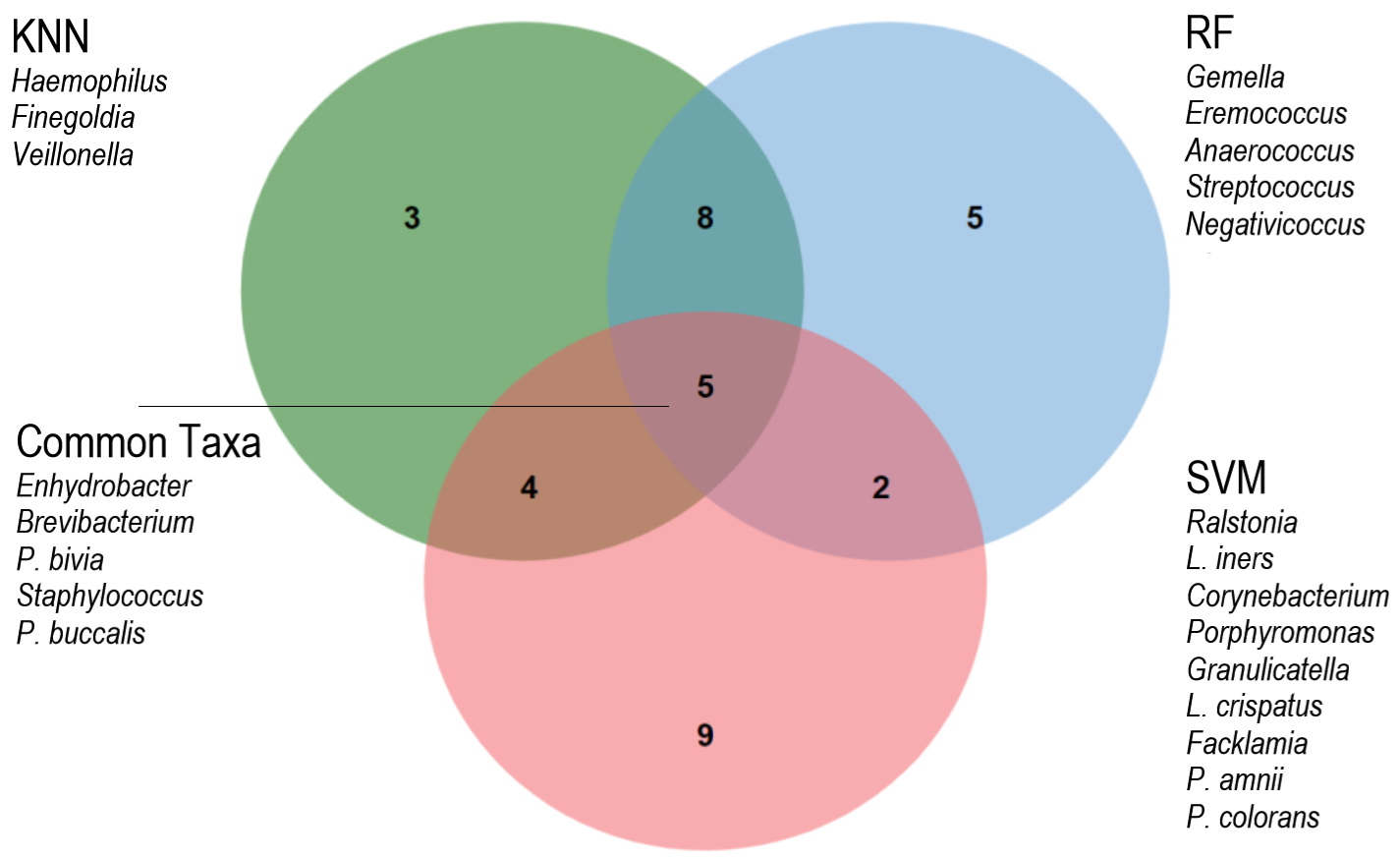

Supplement: Supplemental Figure 5 — Venn Diagram of 20 top-ranked glans/coronal sulcus taxa predicting Bacterial vaginosis, by machine learning classifier. This Venn diagram shows the overlap of the top 20 important variables from each classifier: KNN, K Nearest Neighbor; RF, Random Forest; and SVM, Support Vector Machine. The unique variables are listed for each classifier and the common taxa across all three are listed as indicated. [file Image_5.PNG]
