# Supplementary material for: The Microbiome Composition of a Man's Penis Predicts Incident Bacterial Vaginosis in His Female Sex Partner With High Accuracy
Source: Front Cell Infect Microbiol. 2020 Aug 4;10:433. doi: 10.3389/fcimb.2020.00433 (PMC7438843; doi:10.3389/fcimb.2020.00433)
Supplement: Supplementary file 8 [file Data_Sheet_3.zip › Table 1 (3).docx]

**Supplemental Table 1. Presence and mean relative abundance of 20 most abundant glans/coronal sulcus taxa by incident Bacterial vaginosis status.**

|  | Presence | | Relative Abundance, % (SD) | |
| --- | --- | --- | --- | --- |
|  | Female partner remained BV negative, N=54  n (%) | Female partner had incident BV, N=24  n (%) | Female partner remained BV negative  N=54 | Female partner had incident BV  N=24 |
| *Corynebaterium* | 54 (100) | 24 (100) | 39.9 (27.2) | 34.2 (28.0) |
| *Staphylococcus* | 52 (96) | 19 (79) | 12.1 (14.2) | 13.2 (17.4) |
| *Finegoldia* | 51 (94) | 23 (96) | 7.98 (12.0) | 9.64 (16.0) |
| *Anaerococcus* | 52 (96) | 24 (100) | 8.58 (9.94) | 7.44 (9.22) |
| *Peptoniphilus* | 46 (85) | 23 (96) | 5.32 (8.23) | 7.70 (9.07) |
| *Ezakiella* | 36 (67) | 21 (88) | 3.12 (7.95) | 7.31 (14.1) |
| *Porphyromonas* | 30 (56) | 17 (71) | 1.40 (4.50) | 3.79 (7.35) |
| *Streptococcus* | 43 (80) | 17 (71) | 3.33 (11.6) | 0.96 (2.37) |
| *Prevotella timonensis* | 24 (44) | 13 (54) | 1.47 (3.89) | 2.26 (4.76) |
| *Eremococcus* | 41 (76) | 19 (79) | 2.05 (2.86) | 1.08 (1.45) |
| *Gardnerella vaginalis* | 12 (22) | 7 (29) | 0.64 (2.35) | 1.45 (4.08) |
| *Veillonella* | 19 (35) | 9 (38)_ | 1.25 (4.46) | 0.80 (2.97) |
| *Prevotella corporis* | 12 (22) | 8 (33) | 1.31 (5.28) | 0.58 (1.25) |
| Corynebacteriaceae | 33 (61) | 14 (58) | 0.93 (2.23) | 0.92 (1.51) |
| *Lactobacillus iners* | 24 (44) | 8 (33) | 0.89 (3.10) | 0.95 (3.54) |
| *Sneathia sanguinegens* | 16 (30) | 10 (42) | 1.34 (5.00) | 0.46 (1.30) |
| *Acinetobacter* | 28 (52) | 10 (42) | 0.78 (2.42) | 0.10 (0.230 |
| *Dialister* | 26 (48) | 11 (46) | 0.36 (0.99) | 0.80 (1.37) |
| *Prevotella buccalis* | 16 (30) | 13 (54) | 0.74 (2.14) | 0.40 (0.75) |
| *Facklamia* | 32 (59) | 13 (54) | 0.35 (1.31) | 0.75 (2.24) |
