# Supplementary material for: The Microbiome Composition of a Man's Penis Predicts Incident Bacterial Vaginosis in His Female Sex Partner With High Accuracy
Source: Front Cell Infect Microbiol. 2020 Aug 4;10:433. doi: 10.3389/fcimb.2020.00433 (PMC7438843; doi:10.3389/fcimb.2020.00433)
Supplement: Supplementary file 8 [file Data_Sheet_3.zip › Table 10.docx]

**Supplemental Table 10.** **Classification performance for prediction of incident Bacterial vaginosis in women by male partner’s meatal microbiome, raw data.**

|  | **Random Forest** | **Support Vector Machine** | **K Nearest Neighbor** | **Voting** |
| --- | --- | --- | --- | --- |
| **Accuracy** | 0.5834 | 0.5916 | 0.6213 | 0.6454 |
| **Specificity** | 0.7209 | 0.6298 | 0.7546 | 0.8684 |
| **Sensitivity** | 0.2766 | 0.5061 | 0.3238 | 0.1480 |
| **Area Under the Curve (AUC)** | 0.4886 | 0.5671 | 0.5080 | 0.5640 |
