# Supplementary material for: The Microbiome Composition of a Man's Penis Predicts Incident Bacterial Vaginosis in His Female Sex Partner With High Accuracy
Source: Front Cell Infect Microbiol. 2020 Aug 4;10:433. doi: 10.3389/fcimb.2020.00433 (PMC7438843; doi:10.3389/fcimb.2020.00433)
Supplement: Supplementary file 8 [file Data_Sheet_3.zip › Table 11.docx]

**Supplemental Table 11.** **Classification performance for prediction of incident Bacterial vaginosis in women by male partner’s glans/coronal sulcus microbiome, raw data.**

|  | **Random Forest** | **Support Vector Machine** | **K Nearest Neighbor** | **Voting** |
| --- | --- | --- | --- | --- |
| **Accuracy** | 0.5474 | 0.5417 | 0.5542 | 0.6226 |
| **Specificity** | 0.6632 | 0.6033 | 0.6492 | 0.8641 |
| **Sensitivity** | 0.3021 | 0.4112 | 0.3559 | 0.1107 |
| **Area Under the Curve (AUC)** | 0.5303 | 0.3358 | 0.5011 | 0.4810 |
