# Supplementary material for: The Microbiome Composition of a Man's Penis Predicts Incident Bacterial Vaginosis in His Female Sex Partner With High Accuracy
Source: Front Cell Infect Microbiol. 2020 Aug 4;10:433. doi: 10.3389/fcimb.2020.00433 (PMC7438843; doi:10.3389/fcimb.2020.00433)
Supplement: Supplementary file 8 [file Data_Sheet_3.zip › Table 2 (1).docx]

**Supplemental Table 2. Presence and mean relative abundance of 20 most abundant meatal taxa by circumcision status.**

|  | Meatal Samples, N=168 | | | |
| --- | --- | --- | --- | --- |
|  | Presence | | Mean Relative Abundance, % (SD) | |
|  | Circumcised  N=99  n (%) | Uncircumcised  N=69  n (%) | Circumcised  N=99  n (%) | Uncircumcised N=69  n (%) |
| *Corynebacterium* | 98 (99) | 67 (97) | 23.3 (21.2) | 9.3 (15.9) |
| *Streptococcus* | 81 (82) | 51 (74) | 10.9 (19.1) | 7.6 (17.7) |
| *Anaerococcus* | 97 (98) | 68 (99) | 6.3 (7.0) | 13.3 (10.6) |
| *Finegoldia* | 97 (98) | 69 (100) | 3.4 (4.9) | 14.4 (14.3) |
| *Lactobacillus iners* | 56 (57) | 45 (65) | 6.8 (15.4) | 7.3 (18.4) |
| *Peptoniphilus* | 86 (87) | 68 (99) | 2.4 (3.5) | 10.0 (9.0) |
| *Staphylococcus* | 96 (97) | 53 (77) | 8.7 (11.7) | 0.86 (1.6) |
| *Ralstonia* | 43 (43) | 41 (59) | 4.1 (10.2) | 3.9 (12.5) |
| *Sneathia sanguinegens* | 44 (44) | 28 (41) | 6.4 (13.8) | 2.4 (6.4) |
| *Ezakiella* | 64 (65) | 59 (86) | 0.92 (2.6) | 6.2 (12.4) |
| *Veillonella* | 55 (56) | 42 (61) | 3.7 (7.7) | 2.0 (5.2) |
| *Gardnerella vaginalis* | 51 (52) | 39 (57) | 2.7 (5.3) | 1.8 (4.5) |
| *Prevotella timonensis* | 47 (47) | 48 (70) | 0.60 (1.6) | 3.3 (6.3) |
| *Porphyromonas* | 55 (56) | 56 (81) | 1.0 (3.2) | 2.8 (4.6) |
| *Granulicatella* | 37 (37) | 25 (36) | 1.7 (6.6) | 0.5 (1.4) |
| *Ureaplasma* | 64 (65) | 40 (58) | 1.4 (3.6) | 0.6 (2.0) |
| *Prevotella amnii* | 24 (24) | 12 (17) | 1.6 (4.7) | 0.34 (1.2) |
| *Hydrotalea* | 39 (39) | 39 (57) | 0.76 (2.0) | 1.5 (5.3) |
| *Mesorhizobium* | 26 (26) | 25 (36) | 0.89 (4.3) | 0.42 (2.5) |
| *Escherichia Shigella* | 23 (23) | 13 (19) | 1.1 (5.9) | 0.05 (0.15) |

SD = Standard Deviation
