# Supplementary material for: The Microbiome Composition of a Man's Penis Predicts Incident Bacterial Vaginosis in His Female Sex Partner With High Accuracy
Source: Front Cell Infect Microbiol. 2020 Aug 4;10:433. doi: 10.3389/fcimb.2020.00433 (PMC7438843; doi:10.3389/fcimb.2020.00433)
Supplement: Supplementary file 8 [file Data_Sheet_3.zip › Table 3.docx]

**Supplemental Table 3. Presence and mean relative abundance of 20 most abundant glans/coronal sulcus taxa by circumcision status.**

|  | Glans/Coronal Sulcus Samples, N=78 | | | |
| --- | --- | --- | --- | --- |
|  | Presence | | Mean Relative Abundance, % (SD) | |
|  | Circumcised  N=52  n (%) | Uncircumcised N=26  n (%) | Circumcised  N=52  n (%) | Uncircumcised N=26  n (%) |
| *Corynebacterium* | 52 (100) | 26 (100) | 50.8 (22.0) | 12.8 (18.0) |
| *Staphylococcus* | 52 (100) | 19 (73) | 17.0 (15.4) | 3.3 (9.6) |
| *Finegoldia* | 49 (94) | 25 (96) | 3.8 (6.4) | 17.9 (17.8) |
| *Anaerococcus* | 50 (96) | 26 (100) | 6.8 (8.1) | 11.2 (11.8) |
| *Peptoniphilus* | 43 (83) | 26 (100) | 1.6 (2.5) | 14.9 (9.3) |
| *Ezakiella* | 34 (65) | 23 (88) | 0.33 (0.70) | 12.6 (14.9) |
| *Streptococcus* | 42 (81) | 18 (69) | 3.8 (11.9) | 0.28 (0.52) |
| *Porphyromonas* | 28 (54) | 19 (73) | 0.16 (0.30) | 6.1 (8.5) |
| *Eremococcus* | 6 (88) | 14 (54) | 2.4 (2.8) | 0.48 (1.2) |
| *Prevotella timonensis* | 15 (29) | 22 (85) | 0.09 (0.23) | 5.0 (6.1) |
| *Veillonella* | 18 (35) | 10 (38) | 1.4 (4.9) | 0.45 (1.1) |
| *Prevotella corporis* | 6 (12) | 14 (54) | 0.004 (0.01) | 3.2 (7.3) |
| *Sneathia sanguinegens* | 16 (31) | 10 (38) | 1.5 (5.1) | 0.15 (0.41) |
| Cornyebacteriaceae | 35 (67) | 12 (46) | 1.17 (2.3) | 0.43 (1.0) |
| *Lactobacillus iners* | 23 (44) | 9 (35) | 1.22 (3.9) | 0.28 (0.86) |
| *Gardnerella vaginalis* | 16 (31) | 3 (12) | 1.3 (3.6) | 0.03 (0.12) |
| *Prevotella buccalis* | 9 (17) | 20 (77) | 0.02 (0.09) | 1.9 (2.8) |
| *Acinetobacter* | 33 (63) | 5 (19) | 0.79 (2.5) | 0.15 (0.40) |
| *Dialister* | 18 (35) | 19 (73) | 0.09 (0.28) | 1.32 (1.7) |
| *Facklamia* | 30 (58) | 15 (58) | 0.47 (1.4) | 0.50 (2.1) |

SD = Standard Deviation
