# Supplementary material for: The Microbiome Composition of a Man's Penis Predicts Incident Bacterial Vaginosis in His Female Sex Partner With High Accuracy
Source: Front Cell Infect Microbiol. 2020 Aug 4;10:433. doi: 10.3389/fcimb.2020.00433 (PMC7438843; doi:10.3389/fcimb.2020.00433)
Supplement: Supplementary file 8 [file Data_Sheet_3.zip › Table 4.docx]

**Supplemental Table 4. Classification performance for prediction of incident Bacterial vaginosis in women by male partner’s glans/coronal sulcus microbiome.**

|  | **Random Forest** | **Support Vector Machine** | **K Nearest Neighbor** | **Voting** |
| --- | --- | --- | --- | --- |
| **Accuracy** | 0.656 | 0.785 | 0.681 | **0.768** |
| **Specificity** | 0.696 | 0.772 | 0.430 | **0.699** |
| **Sensitivity** | 0.611 | 0.800 | 0.966 | **0.851** |
| **Area Under the Curve (AUC)** | 0.701 | 0.865 | 0.870 | **0.869** |
