# Supplementary material for: The Microbiome Composition of a Man's Penis Predicts Incident Bacterial Vaginosis in His Female Sex Partner With High Accuracy
Source: Front Cell Infect Microbiol. 2020 Aug 4;10:433. doi: 10.3389/fcimb.2020.00433 (PMC7438843; doi:10.3389/fcimb.2020.00433)
Supplement: Supplementary file 8 [file Data_Sheet_3.zip › Table 6.docx]

**Supplemental Table 6. Classification performance for prediction of incident Bacterial vaginosis in women by male partner’s meatal microbiome: Sensitivity analysis excluding observations in which the female partner had intermediate Nugent score (4-6) at baseline.**

|  | **Random Forest** | **Support Vector Machine** | **K Nearest Neighbor** | **Voting** |
| --- | --- | --- | --- | --- |
| **Accuracy** | 0.8137 | 0.9078 | 0.7847 | 0.8806 |
| **Specificity** | 0.8170 | 0.8840 | 0.5913 | 0.8303 |
| **Sensitivity** | 0.8103 | 0.9332 | 0.9897 | 0.9339 |
| **Area Under the Curve (AUC)** | 0.8837 | 0.9734 | 0.9543 | 0.9690 |
