# Supplementary material for: The Microbiome Composition of a Man's Penis Predicts Incident Bacterial Vaginosis in His Female Sex Partner With High Accuracy
Source: Front Cell Infect Microbiol. 2020 Aug 4;10:433. doi: 10.3389/fcimb.2020.00433 (PMC7438843; doi:10.3389/fcimb.2020.00433)
Supplement: Supplementary file 8 [file Data_Sheet_3.zip › Table 7.docx]

**Supplemental Table 7. Classification performance for prediction of incident Bacterial vaginosis in women by male partner’s glans/coronal sulcus microbiome: Sensitivity analysis excluding observations in which the female partner had intermediate Nugent score (4-6) at baseline.**

|  | **Random Forest** | **Support Vector Machine** | **K Nearest Neighbor** | **Voting** |
| --- | --- | --- | --- | --- |
| **Accuracy** | 0.7826 | 0.8575 | 0.7154 | 0.8356 |
| **Specificity** | 0.7659 | 0.7586 | 0.4296 | 0.7414 |
| **Sensitivity** | 0.7990 | 0.9544 | 0.9954 | 0.9404 |
| **Area Under the Curve (AUC)** | 0.8716 | 0.9405 | 0.8993 | 0.9465 |
