# Supplementary material for: The Microbiome Composition of a Man's Penis Predicts Incident Bacterial Vaginosis in His Female Sex Partner With High Accuracy
Source: Front Cell Infect Microbiol. 2020 Aug 4;10:433. doi: 10.3389/fcimb.2020.00433 (PMC7438843; doi:10.3389/fcimb.2020.00433)
Supplement: Supplementary file 8 [file Data_Sheet_3.zip › Table 9.docx]

**Supplemental Table 9. Variable Importance Ranking by Classifier and for Voting from Glans/Coronal Sulcus Samples Excluding Observations in which Female Sex Partner had Intermediate Nugent Score at Baseline: Top 20 Taxa by Voting.**

| **Variable** | **K-Nearest Neighbor Rank** | **Random Forest Rank** | **Support Vector Machine Rank** | **Voting Rank** |
| --- | --- | --- | --- | --- |
| *Ruminococcaceae ucg014* | 1 | 1 | 1 | 1 |
| *Rothia* | 2 | 2 | 5 | 2 |
| *Kocuria* | 4 | 3 | 13 | 3 |
| *Brevibacterium* | 5 | 9 | 7 | 4 |
| *Enhydrobacter* | 6 | 24 | 4 | 5 |
| *Megasphaera* | 8 | 8 | 22 | 6 |
| *Peptostreptococcus* | 12 | 19 | 11 | 7 |
| Peptostreptococcaceae | 20 | 4 | 20 | 8 |
| *Prevotella disiens* | 7 | 34 | 3 | 9 |
| *Prevotella bivia* | 24 | 13 | 8 | 10 |
| *Acinetobacter* | 3 | 47 | 2 | 11 |
| *Porphyromonas* | 11 | 22 | 19 | 12 |
| *Gardnerella vaginalis* | 10 | 12 | 34 | 13 |
| *Negativicoccus* | 22 | 5 | 32 | 14 |
| *Prevotella timonensis* | 32 | 15 | 12 | 15 |
| *Eremococcus* | 31 | 14 | 17 | 16 |
| *Prevotella buccalis* | 19 | 39 | 6 | 17 |
| *Gemella* | 14 | 29 | 26 | 18 |
| *Prevotella corporis* | 16 | 38 | 15 | 19 |
| *Ezakiella* | 15 | 48 | 9 | 20 |

**Legend:** This table shows the 20 top-ranked taxa by voting, and their variable importance ranking according to each of the other classifiers. The voting importance ranking is determined by averaging the rank of across the three classifiers. We use conditional formatting (Excel) to facilitate reading ranks across the three classifiers, whereby red represents taxa ranked with higher importance and blue represents taxa that are ranked with lower importance. These are the results of sensitivity analyses excluding observations in which the woman had intermediate Nugent score (4-6) at baseline.
