# Supplementary material for: The Microbiome Composition of a Man's Penis Predicts Incident Bacterial Vaginosis in His Female Sex Partner With High Accuracy
Source: Front Cell Infect Microbiol. 2020 Aug 4;10:433. doi: 10.3389/fcimb.2020.00433 (PMC7438843; doi:10.3389/fcimb.2020.00433)
Supplement: Supplementary file 9 [file Table_1.DOCX]

library(randomForest)

library(e1071)

library(ROCR)

library(caret)

library(kknn)

#voting alrorithm for Random Forest, SVM, and KNN

myvoting_3_smote_gcss<-function(k,data){

data=smote_gcss

#stratified cross validation

data_pos<-subset(data,bv_index=="1")

data_neg<-subset(data,bv_index=="0")

data_pos$id <- sample(1:k, nrow(data_pos), replace = TRUE)

data_neg$id<- sample(1:k, nrow(data_neg), replace = TRUE)

list <- 1:k

prediction <- data.frame()

testsetCopy <- data.frame()

for (i in 1:k){

trainingset1 <- subset(data_pos, id %in% list[-i])

trainingset2 <- subset(data_neg, id %in% list[-i])

trainingset<-rbind(trainingset1,trainingset2)

testset1 <- subset(data_pos, id %in% c(i))

testset2 <- subset(data_neg, id %in% c(i))

testset<-rbind(testset1,testset2)

# run a random forest model

x<-trainingset[,3:52]

y<-trainingset[,2]

rm <- tune.randomForest(x,y, ntree=c(10,25,50),nodesize=c(5,15,20))

bestrm=rm$best.model

# run a svm

tune.out=tune(svm,x, y, kernel="radial", cost=c(10,25,50,75,100),probability=TRUE)

bestmod=tune.out$best.model

# remove response column, pred using random forest

temp1 <- as.data.frame(predict(bestrm, testset[,3:52]))

prob1 <- as.data.frame(predict(bestrm, testset[,3:52],type="prob"))

# remove response column, pred using svm

temp22 <- predict(bestmod, testset[,3:52],probability=TRUE)

temp2<-as.data.frame(temp22)

prob2<-attr(temp22,"probabilities")

#remove response column, pred using knn

temp4<-kknn(bv_index~., distance = 1,trainingset[,2:52],kernel = "triweight",testset[,2:52],k=15)

pred4<-temp4$fitted.values

prob4<-temp4$prob[,2]

# voting

comb<-as.matrix(cbind(temp1,temp2,pred4))

colnames(comb)<-NULL

temp<-as.data.frame(apply(comb,1,function(x) names(which.max(table(x)))))

colnames(temp)<-NULL

comb1<-as.matrix(cbind(prob1[,2],prob2[,1],prob4))

colnames(comb1)<-NULL

prob<-as.data.frame(rowMeans(comb1))

final<-cbind(temp,prob)

prediction <- rbind(prediction, final)

testsetCopy <- rbind(testsetCopy, as.data.frame(testset[ , 2]))

}

# add predictions and actual Sepal Length values

result<- cbind(prediction, testsetCopy)

names(result) <- c("predicted","yes", "actual")

result$Difference <- abs(as.numeric(result$actual) - as.numeric(result$predicted))

acucy<-1-sum(result$Difference)/nrow(result)

pred<-prediction(as.numeric(result$yes),result$actual)

auc.perf=performance(pred,measure="auc")

auc<-as.numeric(auc.perf@y.values)

predict<-factor(result$predicted)

truth<-factor(result$actual)

predtable<-table(factor(predict,levels=c("1","0")),factor(truth,level=c("1","0")))

confz<-confusionMatrix(predtable,mode="everything")

sens<-confz$byClass[1]

spec<-confz$byClass[2]

final<-rbind(acucy,spec,sens,auc)

return(final)

}

# Random forest only

myRM<-function(k,data)

{

data_pos<-subset(data,bv_index==1)

data_neg<-subset(data,bv_index==0)

data_pos$id <- sample(1:k, nrow(data_pos), replace = TRUE)

data_neg$id<- sample(1:k, nrow(data_neg), replace = TRUE)

list <- 1:k

prediction <- data.frame()

testsetCopy <- data.frame()

for (i in 1:k){

trainingset1 <- subset(data_pos, id %in% list[-i])

trainingset2 <- subset(data_neg, id %in% list[-i])

trainingset<-rbind(trainingset1,trainingset2)

testset1 <- subset(data_pos, id %in% c(i))

testset2 <- subset(data_neg, id %in% c(i))

testset<-rbind(testset1,testset2)

# run a random forest model

x<-trainingset[,-1]

y<-trainingset[,1]

rm <- tune.randomForest(x,y, ntree=c(10,25,50),nodesize=c(5,15,20))

bestrm=rm$best.model

# remove response column, pred using RF

temp1 <- as.data.frame(predict(bestrm, testset[,-1]))

prob1 <- as.data.frame(predict(bestrm, testset[,-1],type="prob"))

temp<-cbind(prob1,temp1)

prediction <- rbind(prediction, temp)

testsetCopy <- rbind(testsetCopy, as.data.frame(testset[ , 1]))

}

result <- cbind(prediction, testsetCopy)

names(result) <- c("no","yes","predicted", "actual")

result$Difference <- abs(as.numeric(result$actual) - as.numeric(result$predicted))

# As an example use Mean Absolute Error as Evalution

err<-1-sum(result$Difference)/nrow(result)

pred<-prediction(as.numeric(result$yes),result$actual)

ROC.perf<-performance(pred,"tpr","fpr")

auc.perf=performance(pred,measure="auc")

auc<-as.numeric(auc.perf@y.values)

predict<-factor(result$predicted)

truth<-factor(result$actual)

predtable<-table(factor(predict,levels=c("1","0")),factor(truth,level=c("1","0")))

confz<-confusionMatrix(predtable,mode="everything")

sens<-confz$byClass[1]

spec<-confz$byClass[2]

final<-rbind(err,spec,sens,auc)

return(final)

}

mySVM<-function(k,data)

{

data_pos<-subset(data,bv_index==1)

data_neg<-subset(data,bv_index==0)

data_pos$id <- sample(1:k, nrow(data_pos), replace = TRUE)

data_neg$id<- sample(1:k, nrow(data_neg), replace = TRUE)

list <- 1:k

prediction <- data.frame()

testsetCopy <- data.frame()

for (i in 1:k)

{

trainingset1 <- subset(data_pos, id %in% list[-i])

trainingset2 <- subset(data_neg, id %in% list[-i])

trainingset<-rbind(trainingset1,trainingset2)

testset1 <- subset(data_pos, id %in% c(i))

testset2 <- subset(data_neg, id %in% c(i))

testset<-rbind(testset1,testset2)

colnum<-ncol(trainingset)

train<-trainingset[,-colnum]

test<-testset[,-colnum]

x<-as.matrix(train[,-1])

y<-as.factor(as.matrix(train[,1]))

tune.out=tune(svm,x, y, kernel="radial", cost=c(10,25,50,75,100),probability=TRUE)

bestmod=tune.out$best.model

# remove response column

temp <- predict(bestmod, test[,-1],decision.value=TRUE,probability=TRUE)

temp1 <- as.data.frame(predict(bestmod, test[,-1]))

prob<-attr(temp,"probabilities")

temp2<-cbind(temp1,prob)

prediction <- rbind(prediction, temp2)

testsetCopy <- rbind(testsetCopy, as.data.frame(test[ , 1]))

}

# add predictions and actual Sepal Length values

result <- cbind(prediction, testsetCopy)

names(result) <- c("predicted","yes","no", "actual")

result$Difference <- abs(as.numeric(result$actual) - as.numeric(result$predicted))

err<-1-sum(result$Difference)/nrow(result)

pred<-prediction(as.numeric(result$yes),result$actual)

auc.perf=performance(pred,measure="auc")

auc<-as.numeric(auc.perf@y.values)

predict<-factor(result$predicted)

truth<-factor(result$actual)

predtable<-table(factor(predict,levels=c("1","0")),factor(truth,level=c("1","0")))

confz<-confusionMatrix(predtable,mode="everything")

acc<-confz$overall[1]

sens<-confz$byClass[1]

spec<-confz$byClass[2]

final<-rbind(err,spec,sens,auc)

return(final)

}

myKNN<-function(k,data)

{

data_pos<-subset(data,bv_index==1)

data_neg<-subset(data,bv_index==0)

data_pos$id <- sample(1:k, nrow(data_pos), replace = TRUE)

data_neg$id<- sample(1:k, nrow(data_neg), replace = TRUE)

list <- 1:k

prediction <- data.frame()

testsetCopy <- data.frame()

for (i in 1:k){

# remove rows with id i from dataframe to create training set

# select rows with id i to create test set

trainingset1 <- subset(data_pos, id %in% list[-i])

trainingset2 <- subset(data_neg, id %in% list[-i])

trainingset<-rbind(trainingset1,trainingset2)

testset1 <- subset(data_pos, id %in% c(i))

testset2 <- subset(data_neg, id %in% c(i))

testset<-rbind(testset1,testset2)

#remove response column, pred using knn

temp2<-kknn(bv_index~., distance = 1,trainingset[,2:52],kernel = "triweight",testset[,2:52],k=15)

pred<-temp2$fitted.values

prob<-temp2$prob[,2]

temp<-cbind(pred,prob)

temp_new<-temp[,1]-1

test<-cbind(temp_new,prob)

prediction <- rbind(prediction, test)

testsetCopy <- rbind(testsetCopy, as.data.frame(testset[ , 1]))

}

# add predictions and actual Sepal Length values

result<- cbind(prediction, testsetCopy)

names(result) <- c("predicted","yes", "actual")

result$Difference <- abs((as.numeric(result$actual)-1) - as.numeric(result$predicted))

err<-1-sum(result$Difference)/nrow(result)

pred<-prediction(as.numeric(result$yes),result$actual)

auc.perf=performance(pred,measure="auc")

auc<-as.numeric(auc.perf@y.values)

predict<-factor(result$predicted)

truth<-factor(result$actual)

predtable<-table(factor(predict,levels=c("1","0")),factor(truth,level=c("1","0")))

confz<-confusionMatrix(predtable,mode="everything")

acc<-confz$overall[1]

sens<-confz$byClass[1]

spec<-confz$byClass[2]

final<-rbind(err,spec,sens,auc)

return(final)

}

#repeat prediction algorithm (voting, random forest, SVM, KNN) for 1000 times

voting_gcss<-matrix(,nrow=4,ncol=1000)

for (j in 1:1000){

temp<-as.data.frame(myvoting_3(10,gcss_smote))

a<-as.numeric(temp[1,])

b<-as.numeric(temp[2,])

c<-as.numeric(temp[3,])

e<-as.numeric(temp[4,])

voting_gcss[1,j]<-a

voting_gcss[2,j]<-b

voting_gcss[3,j]<-c

voting_gcss[4,j]<-e

}
